# Antiproliferative Effects of *Naja anchietae* and *Naja senegalensis* Venom Peptides on Glioblastoma Cell Lines

**DOI:** 10.3390/toxins16100433

**Published:** 2024-10-10

**Authors:** Yasmine Boughanmi, Caroline Berenguer-Daizé, Marielle Balzano, Hend Mosrati, Maxime Moulard, Pascal Mansuelle, Patrick Fourquet, Franck Torre, Harold de Pomyers, Didier Gigmes, Lhoucine Ouafik, Kamel Mabrouk

**Affiliations:** 1 Aix Marseille University, Institut de Chimie Radicalaire UMR 7273, 13397 Marseille, France; yasmine.boughanmi@etu.univ-amu.fr (Y.B.); mosrati_hend@yahoo.fr (H.M.); didier.gigmes@univ-amu.fr (D.G.); 2 Latoxan, 26800 Portes-lès-Valence, France; harold.pomyers@latoxan.com; 3 Aix-Marseille University, INP—Institute of Neuropathophysiology—UMR 7051 CNRS, 13005 Marseille, France; caroline.berenguer@univ-amu.fr (C.B.-D.); lhoucine.ouafik@univ-amu.fr (L.O.); 4 BioCytex 140 Chemin de l’Armée d’Afrique, 13010 Marseille, France; marielle.balzano@biocytex.fr (M.B.); maxime.moulard@biocytex.fr (M.M.); 5 Proteomics Platform, Marseille Proteomics (MaP), Institut de Microbiologie de la Méditerranée (IMM), FR 3479, CNRS, 13009 Marseille, France; pmansuelle@imm.cnrs.fr; 6 Aix-Marseille University, INSERM, CNRS, Institut Paoli-Calmettes, CRCM, Marseille Proteomics, 13009 Marseille, France; patrick.fourquet@inserm.fr; 7 Aix-Marseille University, Institut Méditerranéen de Biodiversité et d’Ecologie (IMBE), Avignon University, CNRS, IRD, 13397 Marseille, France; franck.torre@univ-amu.fr

**Keywords:** venom, *Naja senegalensis*, *Naja anchietae*, peptide, cobra cytotoxin, antiproliferative activity, apoptosis, necrosis

## Abstract

This study explores the potential of natural bioactive peptides from animal venoms as targeted anti-cancer agents with reduced toxicity. Initially, we screened a broad collection of animal venoms for their antiproliferative activity against cancer cell lines. From this collection, we selected venoms from *Naja anchietae* and *Naja senegalensis* due to their promising activity. Utilizing reverse- phase high-performance liquid chromatography (RP HPLC), mass spectrometry (MALDI-TOF MS and MALDI-TOF TOF MSMS), and Edman degradation sequencing, we isolated and characterized three peptides named CTNanc1, CTNanc2, and CTNanc3 from *Naja anchietae*, and three others named CTNsen1, CTNsen2, and CTNsen3 from *Naja senegalensis*, each with a molecular weight of around 7 kDa. These purified peptides demonstrated inhibition of U87 glioblastoma cell proliferation, but not of U251 and T98G cells, in cell viability assays. To assess the impact of these treatments on cell viability, apoptosis, and necrosis, flow cytometry assays were conducted on U87 cells at 72 h. The results showed a decrease in cell viability and an increase in dead cells, suggesting that the treatments not only promote apoptosis, but may also lead to increased necrosis or late-stage apoptosis as the exposure time increases. These findings suggest that these peptides could be developed as leads for cancer therapy.

## 1. Introduction

Over the past three decades, cancer cases have continued to rise. The five-year relative survival rate for all types of cancer has also increased, partly due to targeted therapies. However, these targeted therapy drugs still have limitations. Therefore, it is crucial to continue developing new biological therapeutic agents that could be used alone or in synergy with FDA-approved drugs to combat cancer. Venoms are a treasure trove of bioactive molecules that have shown potential in treating several diseases, including cancer.

Cytotoxins (CTX), major components of cobra venom, account for approximately 40 to 60% of the total protein content within these venoms. These molecules are typically composed of 59 to 62 amino acids and have a molecular weight ranging between 6 and 9 kDa. The amino acid sequences of these cytotoxins generally share over 60% homology yet exhibit a diverse array of activities. This suggests that even minor differences in amino acid sequences can significantly influence toxin interactions, highlighting the complex nature of these biomolecules [1,2]. The UniProt protein database records over a hundred sequences of CTXs, primarily identified in venoms from snakes of the *Naja* genus, commonly known as cobras. These sequences were discovered predominantly in cobra venom, emphasizing the biological significance and diversity of these toxins [3].

Structurally, CTXs are characterized by their three-dimensional arrangement consisting of five antiparallel β-sheets, which form three functional hydrophobic loops. This distinct structure categorizes them within the three-finger toxin (3FTx) family. The stability of this structure is largely due to the presence of four disulfide bridges that form a robust hydrophobic core, essential for the structural integrity of the proteins [4,5].

The ends of loops I to III in cytotoxins are predominantly composed of hydrophobic residues, which confer amphiphilic properties to these molecules. Around this hydrophobic zone lies a belt of charged residues, dominated by lysine amino acids. This unique structural feature of the molecules, combining hydrophobic and cationic regions, plays a crucial role in their interaction with cellular membranes [6,7]. Notably, loop III exhibits more pronounced structural stability compared to the other loops, starting with the residues located between positions 40 and 45, corresponding to the standard sequence of a CTX composed of 60 amino acids. The residues from 46 to 49, located at the end of loop III, form a type I turn in the β-structure of the protein. Continuing the loop structure, residues from 49 to 54 associate with those from 20 to 26 to establish an antiparallel β-strand. This configuration is of major interest as it plays a key role in the stabilization and functionality of the protein. 

Moving to the cellular impact of cytotoxins, the mechanisms through which they induce cellular responses are diverse and complex. Notably, CTXs have been shown to trigger apoptosis through multiple pathways. For example, CTX3 from *Naja atra* venom activates apoptosis in human myeloid leukemia U937 cells through a cascade involving Ca^2+^, protein phosphatase 2A (PP2A), and AMP-activated protein kinase (AMPK), demonstrating the intricacy of intracellular signaling pathways affected by these toxins. Moreover, CTX1 from the same venom species further illustrates this complexity by inducing the transcription of genes associated with the Fas cell death pathway via a sequence of interactions starting from Ca^2+^ influx, followed by activation of NOX4, and resulting in increased ROS production that activates the p38 MAPK pathway. This detailed cascade of events underscores the cytotoxins’ potential as targets for therapeutic interventions, particularly in the context of diseases characterized by deregulated apoptosis mechanisms [4,8]. Currently, there are no cytotoxins from cobra venom that have progressed to clinical trials or are available on the market specifically for cancer treatment. However, studies continue to explore the anticancer potential of these cytotoxins. 

Research on the application of cytotoxins for glioblastoma treatment is notably sparse, with few studies available in the current literature. Given the highly aggressive nature of glioblastoma, there is a critical need for novel therapeutic approaches. To address this gap, we isolated six distinct peptides from the venoms of two *Naja* species and systematically investigated their antiproliferative activities. Our preliminary results provide initial insights into the potential efficacy of these venom-derived peptides against glioblastoma.

## 2. Results and Discussion

**Figure A1:**
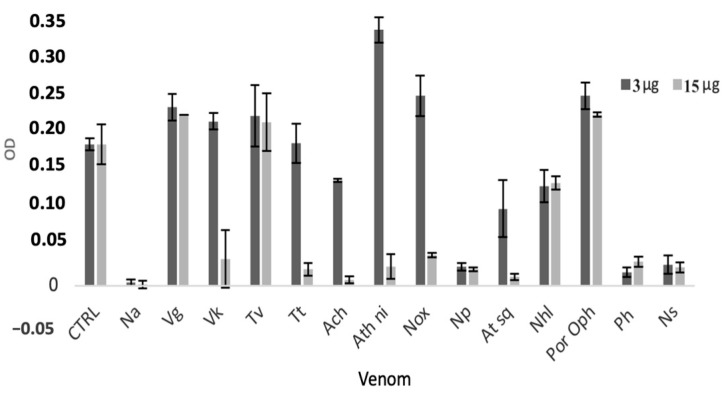
Antiproliferative activity of different animal venoms. These results were obtained using an MTT assay on MCF7 cells after 72 h of treatment with 3 and 15 µg/mL. The OD was measured at 570 nm after 72 h of treatment. The abbreviations include Na: *Naja anchietae*; Vg: *Viridovipera gumprechti*; Vk: *Vipera kaznakovi*; Tv: *Trimeresurus vogeli*; Tt: *Trimeresurus trigonocephalus*; Ach: *Atheris chlorechis*; Ath ni: *Atheris nitschei nitschei*; Nox: *Naja oxiana*; Np: *Naja philippinensis*; At sq: *Atheris squamigera*; Nhl: *Naja haje legionis*; Por Oph: *Porthidium ophryomegas;* Ph: *Parias hageni*; Ns: *Naja senegalensis*.

We screened nearly 40 different animal venoms to evaluate their potential anticancer properties. The venoms were solubilized at a concentration of 1.5 mg/mL in water. The antiproliferative activity of these venoms was tested at two concentrations, 3 µg and 15 µg, against MCF-7 cells (Appendix A, Figure A1). From this group, two highly active venoms, previously undescribed in the literature, *Naja anchietae* and *Naja senegalensis*, were selected and subjected to further fractionation and subfractionation using RP-HPLC to isolate and identify the active components responsible for the observed biological activities. 

### 2.1. Physicochemical Characterization

#### 2.1.1. Purification of CTNs

**Figure 1:**
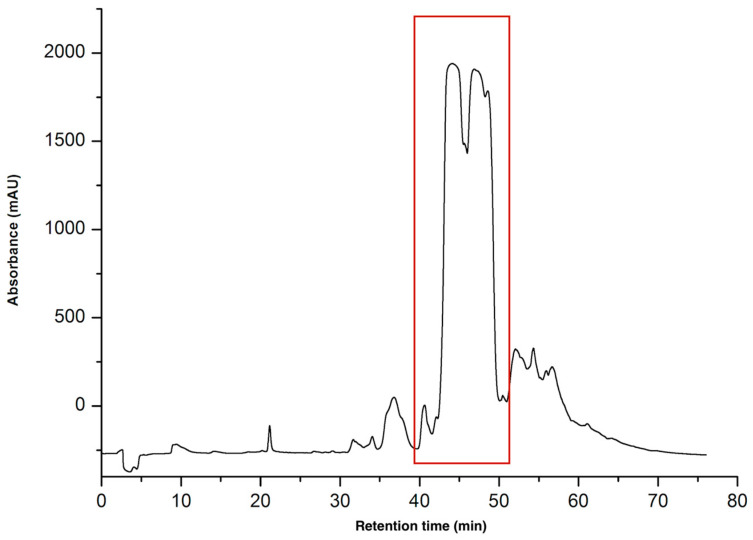
Preparative HPLC chromatogram of *Naja anchietae* venom using gradient 0–70% for 70 min at 214 nm a C18 100 Å column. Fractions were collected at intervals of every 0.1 min, corresponding to a flow rate of 6 mL per minute. The region containing the active peptides is highlighted with a red square in the figure. These peptides were eluted between 40 and 52 min, which corresponds to an elution percentage of approximately 30%.

The venoms of *Naja anchietae* and *Naja senegalensis* were subjected to purification via reverse-phase high-performance liquid chromatography (RP-HPLC). The initial gradient applied was from 0% to 70% solvent B over 70 min, equivalent to a gradient slope of 1% solvent B per minute, utilizing a C18 column with a 100 Å pore size (Figure 1). 

**Figure 2:**
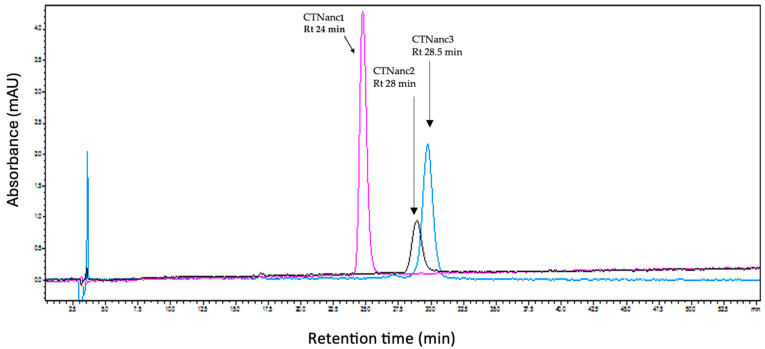
Analytical HPLC chromatogram of the isolated peptides from *Naja anchietae* at 214 nm on a C18 100 Å column.

Three peptides were isolated from this region (Figure 1), designated as CTNanc1, CTNanc2, and CTNanc3. These peptides were characterized using analytical RP-HPLC under a gradient of 20–50% solvent B over 60 min at a flow rate of 1 mL per minute. Detection was carried out at wavelengths of 214 nm and 280 nm. The profile of the homogeneous products is presented in the Figure 2.

**Figure 3:**
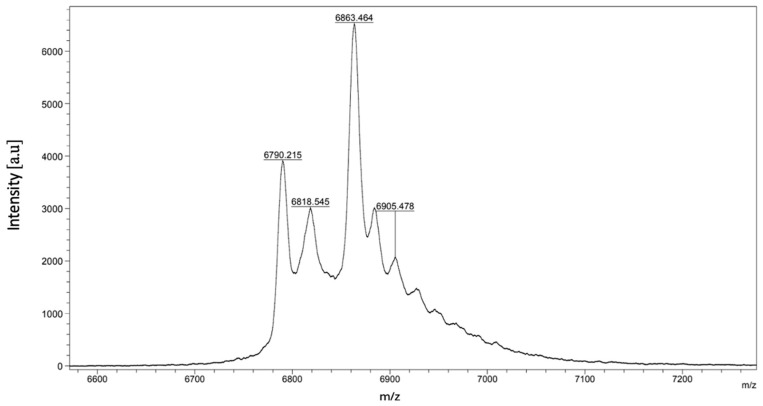
Mass spectrum of CTNanc3 using MALDI-ToF.

As illustrated in Figure 2, the peptides CTNanc2 and CTNanc3 exhibit very similar hydrophobic properties, with retention times differing by only 0.5 min. This similarity has adversely affected the yield of these two peptides. Interestingly, despite the peptide CTNanc3 appearing highly homogeneous in the analytical RP-HPLC profile as shown in the figure, it presented a complex and contaminated mass spectrum (Figure 3). 

**Figure 4:**
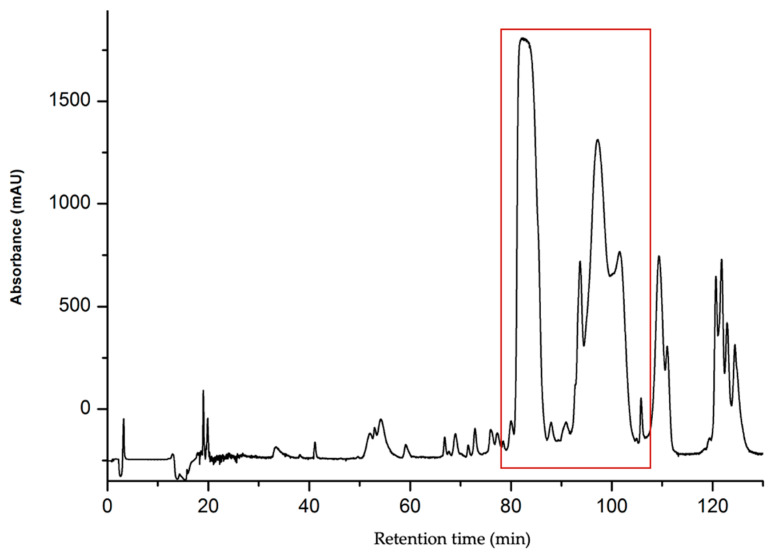
Preparative HPLC chromatogram of *Naja anchietae* venom using gradient 20–60% for 120 min at 214 nm on a C18 300 Å column. Fractions were collected at intervals of every 0.1 min, corresponding to a flow rate of 6 mL per minute. The region containing the active peptides is highlighted with a red square in the figure. These peptides were eluted between 80 and 120 min, which corresponds to an elution percentage of approximately 37%.

Consequently, the purification protocol was adjusted to employ a more gradual gradient of 20% to 50% solvent B over 120 min, corresponding to a gradient slope of 0.25% solvent B per minute. This adjustment was implemented on a Phenomenex Jupiter 300 Å, 5 µm C18 column (250 mm × 21.2 mm), featuring larger pore sizes (Figure 4). This modification led to significantly improved resolution, thereby enhancing the homogeneity and yield of all peptides isolated from these venoms. Indeed, it is believed that materials with wider pores more effectively facilitate the entry of larger proteins into the porous matrix and enhance the partitioning between phases [9]. 

This new strategy enabled the successful purification of the three peptides, with their purity confirmed by mass spectrometry, as demonstrated in the spectra presented in the following section.

**Figure 5:**
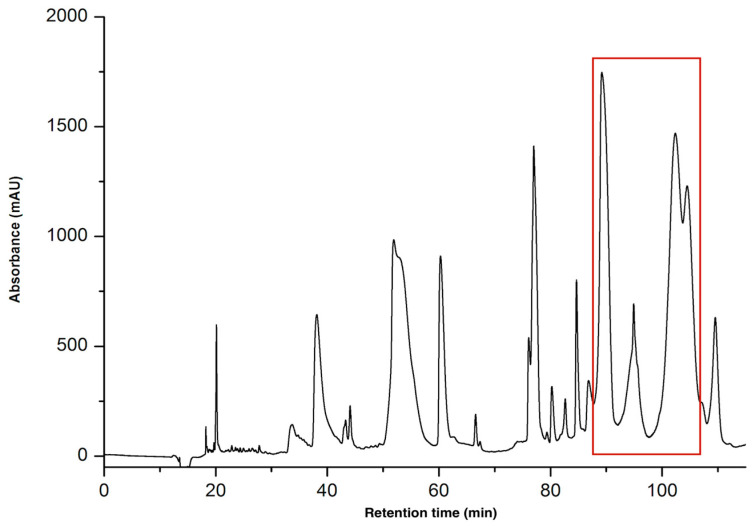
Preparative HPLC chromatogram of *Naja senegalensis* venom using gradient 20–60% for 120 min at 214 nm on a C18 300 Å column. Fractions were collected at intervals of every 0.1 min, corresponding to a flow rate of 6 mL per minute. The region containing the active peptides is highlighted with a red square in the figure. These peptides were eluted between 90 and 120 min, which corresponds to an elution percentage of approximately 40%.

**Figure 6:**
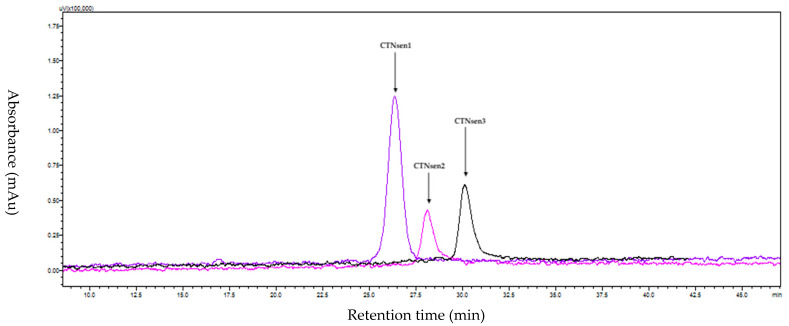
Analytical HPLC chromatogram of the isolated peptides from *Naja senegalensis* at 214 nm on a C18 100 Å column.

Similarly, the venom of *Naja senegalensis* was purified according to this protocol, as depicted in the Figure 5 and Figure 6.

#### 2.1.2. MALDI-TOF Mass Spectrometry

**Figure 7:**
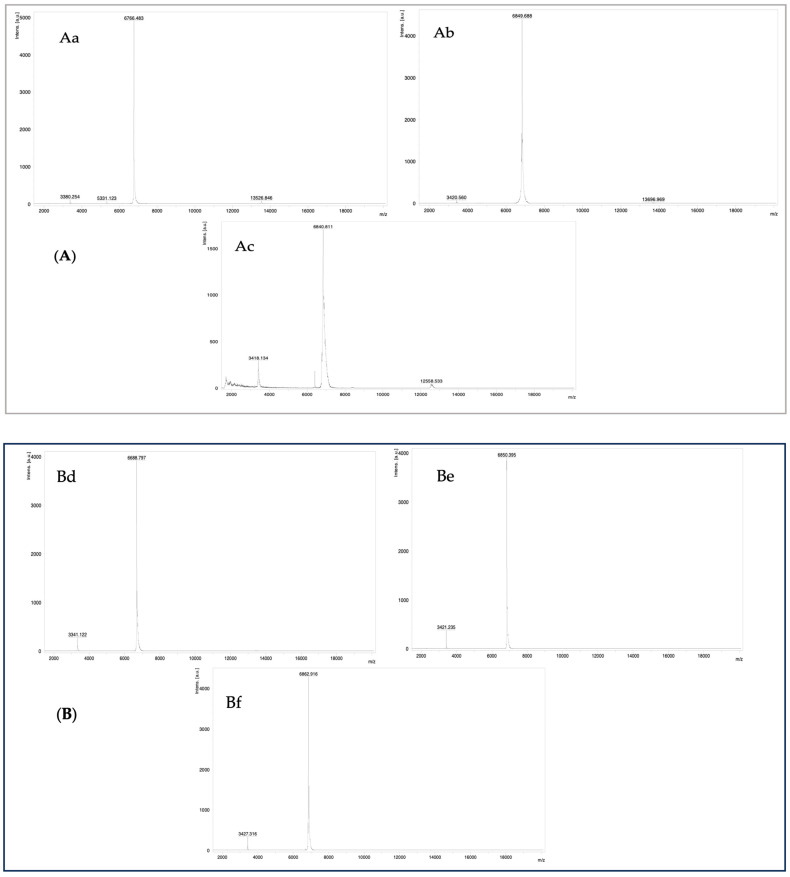
Mass spectrum of peptides CTNsen1 (Aa), CTNsen2 (Ab), CTNsen3 (Ac), CTNanc1 (Bd), CTNanc2 (Be), and CTNanc3 (Bf) using MALDI-ToF.

Active peaks isolated and purified from the venoms of *Naja anchietae* and *Naja senegalensis* were characterized using analytical high-performance liquid chromatography (HPLC). Homogeneous molecules identified via HPLC were subsequently analyzed by matrix-assisted laser desorption/ionization time-of-flight (MALDI-TOF) mass spectrometry. This analysis revealed that the peaks designated as CTNsen1 (Aa), CTNsen2 (Ab), CTNsen3 (Ac), CTNanc1 (Bd), CTNanc2 (Be), and CTNanc3 (Bf) had molecular weights of [M + H] 6766.4 Da, 6849.6 Da, 6840.8 Da, 6688.7 Da, 6850.4 Da, and 6862.9 Da, respectively, as shown in Figure 7.

#### 2.1.3. N-Terminal Edman Sequencing

Peptides from *Naja anchietae* and *Naja senegalensis* were subjected to reduction with dithiothreitol (DTT) and alkylation with 4-vinylpyridine (4VP), as detailed in the Materials and Methods section. Subsequently, these peptides underwent N-terminal Edman sequencing. To identify homologous sequences, a BLAST search was conducted to determine the best alignment scores. The results of the sequencing and the alignments are summarized in the table below. 

All overall repetitive yields exceed 90%, confirming the efficiency and precision of the sequencing performed. Beyond the 50th cycle, the detected PTH amounts decreased due to carryover effects. 

The missing portion of CTNsen1 was determined using MALDI TOF-TOF MS/MS following digestion with the AspN enzyme on the reduced and alkylated peptide. This analysis successfully identified the following C-terminal sequence: ‘DV(PEC)PKNSALVKYV(PEC)(PEC)NT(D)’, as shown in Table 1.

CTNsen1, CTNanc1, and CTNanc2 demonstrated a 100% sequence identity with previously described cytotoxins, indicating that these are well-conserved within the toxin literature. However, CTNsen2, CTNsen3, and CTNanc3 exhibited 98.33% similarity to known cytotoxins, as determined by BLAST analysis. The minor sequence variations identified suggest that these cytotoxins may represent novel variants. These distinctions could potentially influence their biological functions and interactions.

**Figure 8:**
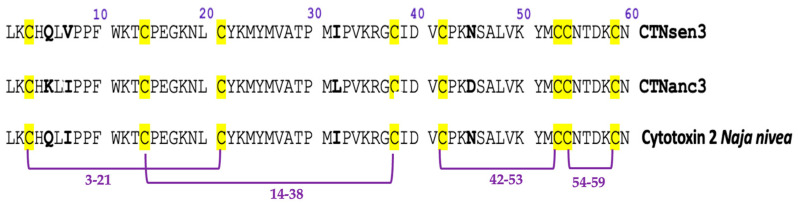
Comparative analysis of CTNsen3, CTNanc3, and Cytotoxin 2 from *Naja nivea* sequences. The differences are in bold and cysteines forming disulfide bridges are highlighted in yellow.

Interestingly, BLAST alignment revealed that the sequences of CTNsen3 and CTNanc3 are both highly similar to the sequence of Cytotoxin 2 from *Naja nivea*. These sequences were compared in Figure 8 to highlight the subtle yet potentially significant variations that distinguish them from the known Cytotoxin 2 sequence.

This cytotoxin family is distinguished by four conserved disulfide bridges that enhance structural stability. The locations of these crucial disulfide linkages are consistent across the family, specifically at positions 3–21, 14–38, 42–53, and 54–59 in the amino acid sequence, underscoring the highly conserved nature of these cytotoxic proteins.

In-depth studies, such as those by Menez et al., have pinpointed key structural features of CTXs, including three critical disulfide bridges named B1 (C1–C3), B2 (C2–C4), and B4 (C7–C8). These bridges, along with adjacent residues, create essential atomic contacts that are crucial for the protein’s functionality. The substitution of cysteine residues with alanines in proteins, resulting in the removal of a specific disulfide bridge, has shown that the elimination of bridges B3 (C5–C6) or B4 drastically alters the three-finger structure of the protein. Conversely, the removal of bridges B1 or B2 does not significantly impact the biological function of the proteins. This discovery underscores the variable importance of disulfide bridges in maintaining the three-dimensional structure of three-finger toxins. While bridges B3 and B4 are essential for the structural integrity required for cytotoxin function, bridges B1 and B2 play a less critical role. Their absence does not prevent the cytotoxin from maintaining a functional conformation [7,10].

Furthermore, the positioning of the Asn60 residue near the terminal cysteine (C8), contributing to a CN motif, is crucial for the conformation of the toxins. This residue, consistently found in this key position across all CTXs, plays a vital role as its side chain forms three hydrogen bonds, thus stabilizing the hydrophobic center of the molecule. Additionally, the transformation of aspartate 57 to asparagine (mutation D57N) has been identified as a disruptive factor for CTX structures in neutral environments. This change weakens interactions within the localized hydrophobic cluster at the C- and N-termini of the molecule, destabilizing the salt bridge between Asp57 and Lys2 [7].

Based on our studies results, CTNsen1 and CTNanc1 are classified as S-type cytotoxins, characterized by their specific structural and functional properties. Conversely, CTNsen2, CTNsen3, CTNanc2, and CTNanc3 are categorized as Type P cytotoxins. Type S cytotoxins present a contrast in their mode of action compared to Type P cytotoxins. While Type P cytotoxins enhance their binding and disruptive capabilities on cellular membranes through an additional binding site associated with Pro 31 (or 30), Type S cytotoxins, characterized by the Ser 29 (or 28) residue, tend to engage in more hydrophilic interactions. This fundamental structural difference influences how these toxins affect cellular membranes: Type P cytotoxins are adept at deeper penetration into the lipid bilayer, enhancing their membrane destabilization effects as observed in the studies conducted by Suzuki-Matsubara et al. and Dubovskii et al., leading to necrosis or apoptosis [11,12]. In contrast, Type S cytotoxins primarily interact through electrostatic attractions facilitated by their charged lysine rich loops and are often studied for their ability to induce programmed cell death (apoptosis) rather than immediate lysis. This results in a milder impact on membrane structures, with interactions largely confined to the surface rather than extensive penetration. This mode of action does not lead to the extensive disruption typical of Type P, reflecting a more nuanced approach to cellular interaction that could have different biological implications [5,13]. Some of the examples of S and P type cytotoxins from *Naja* species are listed in the Table 2 below.

In the scientific literature, numerous cytotoxins derived from snake venoms have been described for their efficacy against various types of cancer and malignancies, such as leukemia, breast cancers, and lung cancers. However, the exploration of snake venom cytotoxins in the context of glioblastoma treatment remains scarce or even non-existent.

An alternative classification of cytotoxins introduced in 2024 by Dubovskii and Utkin provides a new approach to understanding their interactions with cellular membranes. This new classification system categorizes cytotoxins based on the presence of key amino acids that significantly influence their membrane activity. According to this scheme, cytotoxins such as CTNsen1, CTNsen2, CTNanc1, CTNanc2, and CTNanc3, which contain the specific residues Pro9, Ser28, and Asn/Asp45, are assigned to Group 1. This group is characterized by a lower membrane disruptive activity, indicating a nuanced mechanism of action where these cytotoxins bind to cell membranes but are less likely to form disruptive pores (1). This result does not align well with our flow cytometry (FACS) findings for the peptides CTNanc1 and CTNanc2 which demonstrated a substantial increase in Propidium iodide (PI)-positive cells. This increase is indicative of late apoptosis or necrosis, suggesting that apoptosis in these cases progresses to a later stage where there is a loss of membrane integrity.

### 2.2. Pharmacological Characterization

Previous research has indicated strong antiproliferative activity of *Naja* venoms in other cancer types, such as MCF-7 [5], which motivated our study in the specific context of glioblastoma. Moreover, cytotoxicity tests to evaluate antiproliferative effects are generally simpler and more straightforward than those required for other tests. Furthermore, in glioblastoma and other aggressive cancers, the rapid proliferation of tumor cells is a major characteristic that contributes to the severity of the disease. Thus, targeting proliferation can have an immediate and measurable therapeutic impact, crucial for developing effective treatments.

#### 2.2.1. In Vitro Cytotoxicity Assay

**Figure 9:**
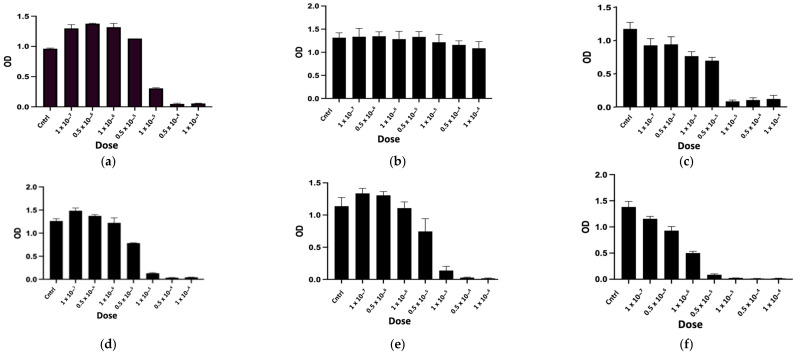
Antiproliferative activity of CTNsen1 (**a**), CTNsen2 (**b**), CTNsen3 (**c**), CTNanc1 (**d**), CTNanc2 (**e**), and CTNanc3 (**f**) from *Naja senegalensis* and *Naja anchietae*, respectively. These results were obtained using an MTT assay on U87 cells after 72 h of treatment with a series of increasing concentrations (1 × 10^−^⁷, 0.5 × 10^−6^, 1 × 10^−^⁶, 0.5 × 10^−5^, 1 × 10^−^⁵, 0.5 × 10^−4^, and 1 × 10^−^⁴ Molar) of peptides. The OD was measured at 570 nm.

**Figure 10:**
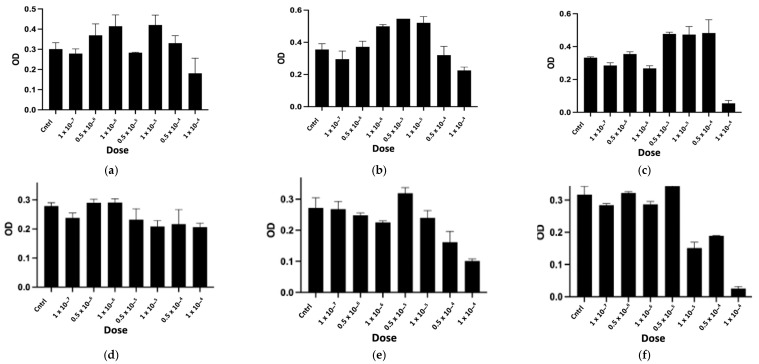
Antiproliferative activity of CTNsen1 (**a**), CTNsen2 (**b**), CTNsen3 (**c**), CTNanc1 (**d**), CTNanc2 (**e**), and CTNanc3 (**f**) from *Naja senegalensis* and *Naja anchietae* respectively. These results were obtained using an MTT assay on U251 cells after 72 h of treatment with a series of increasing concentrations (1 × 10^−^⁷, 0.5 × 10^−6^, 1 × 10^−^⁶, 0.5 × 10^−5^, 1 × 10^−^⁵, 0.5 × 10^−4^, and 1 × 10^−4^ Molar) of peptides. The OD was measured at 570 nm.

**Figure 11:**
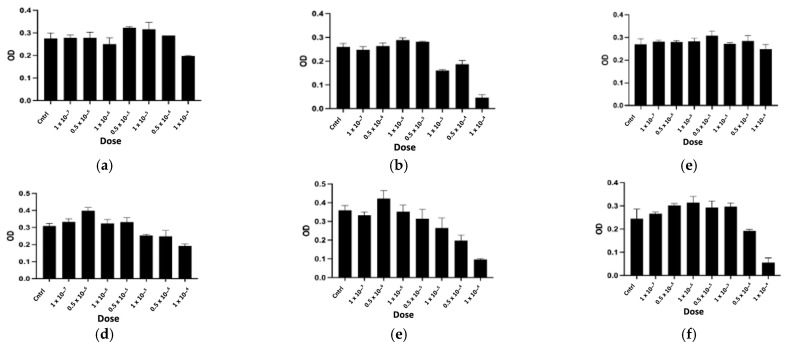
Antiproliferative activity of CTNsen1 (**a**), CTNsen2 (**b**), CTNsen3 (**c**), CTNanc1 (**d**), CTNanc2 (**e**), and CTNanc3 (**f**) from *Naja senegalensis* and *Naja anchietae*, respectively. These results were obtained using an MTT assay on T98G cells after 72 h of treatment with a series of increasing concentrations (1 × 10^−^⁷, 0.5 × 10^−6^, 1 × 10^−^⁶, 0.5 × 10^−5^, 1 × 10^−^⁵, 0.5 × 10^−4^, and 1 × 10^−^⁴ Molar) of peptides. The OD was measured at 570 nm.

**Figure 12:**
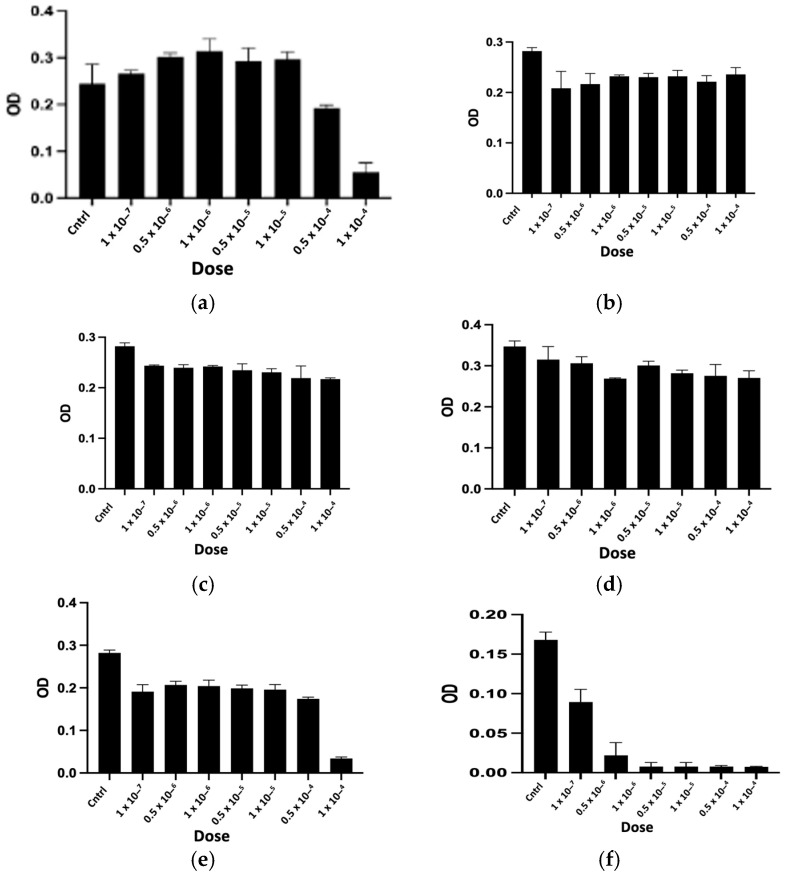
Cytotoxic activity of CTNsen1 (**a**), CTNsen2 (**b**), CTNsen3 (**c**), CTNanc1 (**d**), CTNanc2 (**e**), and CTNanc3 (**f**) from *Naja senegalensis* and *Naja anchietae*, respectively. These results were obtained using an MTT assay on HUVEC cells after 72 h of treatment with a series of increasing concentrations (1 × 10^−^⁷, 0.5 × 10^−6^, 1 × 10^−^⁶, 0.5 × 10^−5^, 1 × 10^−^⁵, 0.5 × 10^−4^, and 1 × 10^−^⁴ Molar) of peptides. The OD was measured at 570 nm.

The purified peptides, named CTNsen1, CTNsen2, CTNsen3, CTNanc1, CTNanc2, and CTNanc3 from *Naja senegalensis* and *Naja anchietae* respectively, were subsequently tested for their antiproliferative effects on several glioblastoma cell lines (U87, U251, and T98G) as well as on primary human umbilical vein endothelial cells (HUVECs). A series of increasing concentrations (1 × 10^−^⁷, 0.5 × 10^−6^, 1 × 10^−^⁶, 0.5 × 10^−5^, 1 × 10^−^⁵, 0.5 × 10^−4^, and 1 × 10^−^⁴ Molar) were used. The dose-response curves for each peptide are depicted in Figure 9, Figure 10, Figure 11 and Figure 12. Antiproliferative assays conducted on U87, U251, and T98G glioblastoma cell lines revealed distinct activities for the peptides derived from *Naja senegalensis* and *Naja anchietae*.

To determine whether the six identified peptides exhibit cytotoxicity on non-tumor cells, we chose to test their effects on HUVEC cells, as illustrated in Figure 12.

The IC50 values obtained from these results were calculated and are presented in Table 3.

Our results revealed notable antiproliferative activity of CTNanc3 against the U87 glioblastoma cell line, exhibiting an IC50 of 5.33 μg/mL. However, this peptide also demonstrated substantial toxicity towards HUVEC endothelial cells with a comparable IC50, suggesting that its effects might largely be attributed to general cytotoxicity rather than selective anticancer activity. CTNanc3 displayed minimal activity against U251 and T98G glioblastoma cell lines, with IC50 values of 416.37 μg/mL and 420.01 μg/mL, respectively, indicating poor efficacy in these models. In contrast, CTNanc1 emerged as the most potent peptide against U87 cells, with an IC50 of 36.41 μg/mL and no cytotoxicity against HUVEC endothelial cells up to a concentration of 1 × 10^−4^ M (686 μg). Interestingly, this activity appears to be U87-selective since CTNanc1 showed little or no activity towards U251 and T98G cell lines even at high concentrations. CTNanc2 displayed considerable activity against the U87 cell line with an IC50 of 38.34 μg/mL. However, it also proved cytotoxic towards HUVEC cells, suggesting a toxicity profile that could limit its therapeutic application despite its efficacy (Figure 9, Figure 10, Figure 11 and Figure 12, Table 3).

Concerning the CTNsen peptides on U87 cell lines: (i) CTNsen1 was active with an IC50 of 54.69 μg/mL, but showed notable cytotoxicity against HUVEC cells; (ii) CTNsen2 showed no antiproliferative activity on U87 cells even at high concentrations, nor cytotoxicity on normal cells; and (iii) CTNsen3 displayed interesting antiproliferative activity against U87, with an IC50 of 45.38 μg/mL, and importantly, no cytotoxicity was observed on HUVEC cells at any concentration tested, underscoring its potential safety for non-cancerous tissues. Lastly, considering the cytotoxicity of all peptides on normal cells, no notable antiproliferative activity on the U251 and T98G lines was observed (Figure 9, Figure 10, Figure 11 and Figure 12, Table 3). Overall, CTNanc1 and CTNsen3 stand out as the most effective and selective peptides against U87 cells; they exhibit good activity without toxicity to non-cancerous cells even at high concentrations. 

The variable sensitivity of different glioblastoma cell lines to these peptides suggests that the molecular environment of each cell line, including the expression of specific receptors, membrane composition, and intracellular pathways, plays a critical role in determining their cytotoxic efficacy. Proteomic analysis of U87 and U251 glioblastoma cell lines reveals distinct differences in their protein expression profiles, reflecting their unique cellular characteristics and responses to environmental conditions. U87 cells, known for robust growth and strong adherence in culture, exhibit higher levels of proteins associated with cellular metabolism, cytoskeletal structure, and stress response pathways. This suggests that U87 cells may have adapted to in vitro conditions by optimizing pathways that support their rapid proliferation and structural integrity. In contrast, U251 and T98G cells display a proteomic profile characterized by elevated expression of proteins involved in DNA repair, transcription regulation, and apoptosis, indicating these cells may prioritize genomic stability and controlled cell death mechanisms to maintain their survival in culture. Additionally, U251 cells show a reduction in certain proteins abundantly expressed in U87 cells, highlighting different regulatory mechanisms and potential vulnerabilities [21,22].

Cytotoxins that showed a high percentage of identity with ours via BLAST display varied anticancer activities according to the literature. Cytotoxin 5 from *Naja haje haje*, as reported by Lafnoune et al. in 2021, comprises about 23% of the proteins in the F7 fraction of the venom and exhibits potent anticancer effects. It significantly reduces the size of hepatocellular carcinoma multicellular tumor spheroids, thus impacting cancer cell proliferation directly. This study primarily characterized the anticancer activities of this fraction without further separation into individual components [23]. Conversely, no anticancer activity has been described for Cytotoxin 5 from *Naja haje annulifera* or for Cytotoxin 1 and Cytotoxin 2 from *Naja annulifera*. However, Cytotoxin 2 from *Naja nivea* shows promising results. According to Ebrahim et al. in 2014, this peptide induces apoptosis in the MCF-7 breast adenocarcinoma cell line, demonstrating a significant decrease in cell viability with an IC50 of approximately 4.18 µg/mL, far lower than the standard drug cisplatin. The peptide initiates a cascade of apoptosis-related intracellular events, including the generation of reactive oxygen species (ROS), disruption of mitochondrial membrane potential, and activation of caspase-9 and caspase-3. This indicates a strong potential for therapeutic application, although Cytotoxin 2 was also tested against the non-tumorigenic MCF 10A cell line with an IC50 of 18.12 ± 2.56 µg/mL (around 26.4 × 10^−4^ M), suggesting its broader cytotoxicity profile [15]. Importantly, no studies have described any positive anticancer activity of these peptides against glioblastoma cells in the literature.

To mitigate the harmful effects of cytotoxins on healthy tissue while still effectively targeting glioblastoma cells, several strategies can be employed. One approach involves the use of specialized delivery systems such as liposomes or nanoparticles, which are designed to direct treatment specifically to cancer cells, thereby reducing unwanted effects on other cells. These carriers can be functionalized with antibodies or ligands that exclusively bind to markers overexpressed on glioblastoma cells, ensuring that the cytotoxin is delivered primarily to the tumor site. For instance, lipid crystal nanoparticles and proliposomes encapsulating temozolomide, a well-established medication for glioblastoma treatment, have been engineered to enhance penetration of the blood–brain barrier, facilitating targeted delivery of this chemotherapeutic agent directly to brain tumor cells, as described by Waghule et al. in 2021 [24]. Another strategy is the modification of cytotoxins through PEGylation or conjugation. For example, conjugated toxins like ANG1005, which consists of paclitaxel conjugated with angiopep, target the LRP-1 receptor that is overexpressed in various cancers, including glioblastoma. This conjugation enhances the drug’s ability to traverse the blood–brain barrier and more effectively target tumor cells, as detailed by Kumthekar et al. in 2020 [25]. Lastly, combination therapy serves as a third strategy. Combining cytotoxins with targeted inhibitors or chemotherapy drugs can synergistically increase cancer cell death and mitigate toxicity to normal cells by lowering the required doses of each agent. This strategy not only amplifies the therapeutic impact but also minimizes the chances of developing resistance to single-agent treatments. For instance, combining bevacizumab, an anti-VEGF antibody, with lomustine is employed in treating recurrent glioblastoma to disrupt the tumor’s blood supply concurrently with a direct cytotoxic attack, enhancing overall tumor control [26].

**Figure 13:**
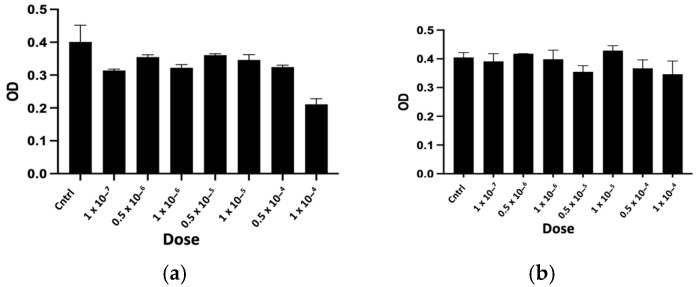
Antiproliferative activity of reduced and alkylated CTNsen1 (**a**) and CTNanc1 (**b**). These results were obtained using an MTT assay on U87 cells after 72 h of treatment with a series of increasing concentrations (1 × 10^−^⁷, 0.5 × 10^−6^, 1 × 10^−^⁶, 0.5 × 10^−5^, 1 × 10^−^⁵, 0.5 × 10^−4^, and 1 × 10^−^⁴ Molar) of peptides. The OD was measured at 570 nm.

Finally, to determine the influence of disulfide bridges on the antiproliferative activity against U87 cells, the peptides CTNanc1 and CTNsen1 were reduced and alkylated before being tested on U87 cells (Figure 13). The disruption of disulfide bonds resulted in a loss of activity compared to the native oxidized peptides, underscoring the importance of these bonds for their antiproliferative activity. Additionally, a comparison of the primary structures of the studied cytotoxins reveals very slight variations in amino acids. Notably, CTNanc1, the most active peptide against the proliferation of U87 cell lines, contains a valine at position 10, unlike the phenylalanine found in other cytotoxins. It also replaces a tyrosine with a phenylalanine at position 25 and features an STSTV sequence from positions 28 to 32. These combined observations suggest that antiproliferative activity depends not only on the three-dimensional structure stabilized by disulfide bonds but also on the potential exposure of certain amino acids critical to this activity.

#### 2.2.2. Flow Cytometry Analysis Results

a. Gating strategy

**Figure 14:**
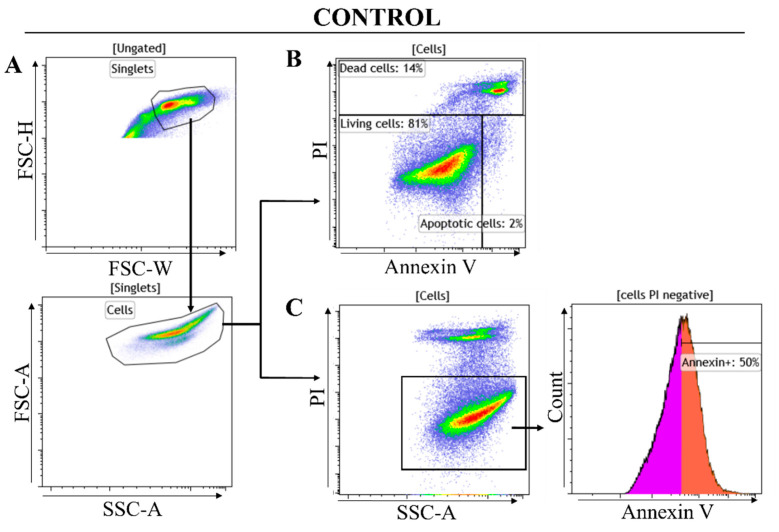
Flow cytometry gating strategy: representative example using untreated U87 cells. Doublets and debris are excluded (**A**); the first gating strategy identifies living cells (PI-negative and Annexin V-negative), apoptotic cells (Annexin V-positive and PI-negative), and dead cells (Annexin V-positive and PI-positive) (**B**); the second gating assesses the intensity of Annexin V fluorescence and the percentage of Annexin V-positive cells, Annexin V-negative and positive cell subsets are colored in pink and orange, respectively (**C**).

The first part of the gating strategy focuses on identifying and isolating viable cells from the total cell population (Figure 14A). This process begins by excluding doublets and aggregates through setting a gate on forward scatter height (FSC-H) versus forward scatter width (FSC-W), ensuring that only individual cells are analyzed. Subsequently, a second gate is applied based on forward scatter area (FSC-A) against side scatter area (SSC-A) to differentiate actual cells from debris and other non-cellular elements. Within this refined population, cells are then analyzed using two gating strategies, as shown in the representative examples for the control in Figure 14B,C.

Cells are analyzed for Annexin V FITC/PI (Figure 14A), which defines the living cells (PI negative and Annexin V negative), the apoptotic cells (Annexin V positive and PI negative), and the dead cells (Annexin V positive and PI positive). The percentages of each cell subset from this analysis are reported in Table 4. The main objective of this gating strategy was to analyze U87 cell death after peptide treatment ([Dead cells] % gated, Table 4). To refine the analysis of the Annexin V-positive population among living cells, the Mean Fluorescence Intensity (MFI) of Annexin V-FITC was recorded (Figure 14B). PI-negative cells are indicative of membrane integrity and, therefore, viability (Figure 14B). Annexin V staining binds to phosphatidylserine on the external membrane of apoptotic cells. The extent of apoptosis within the viable cell subset correlates with the Annexin V fluorescence intensity (MFI). In the histogram from the control, a threshold of positivity was arbitrarily set at 50% to separate the Annexin V-negative and positive cell subsets. The percentage of Annexin V-positive gated cells and the Annexin V fluorescence intensity from the PI-negative (viable) cells are reported in Table 5. The comparison of the MFI and percentage of gated Annexin V-positive cells to the control is correlated with an increase in the apoptosis rate.

Cells exhibiting Annexin V positivity are identified as early apoptotic since they are undergoing apoptosis but have not yet lost their membrane integrity to the extent that would permit PI staining.

b. Cytotoxicity analysis by flow cytometry

**Figure 15:**
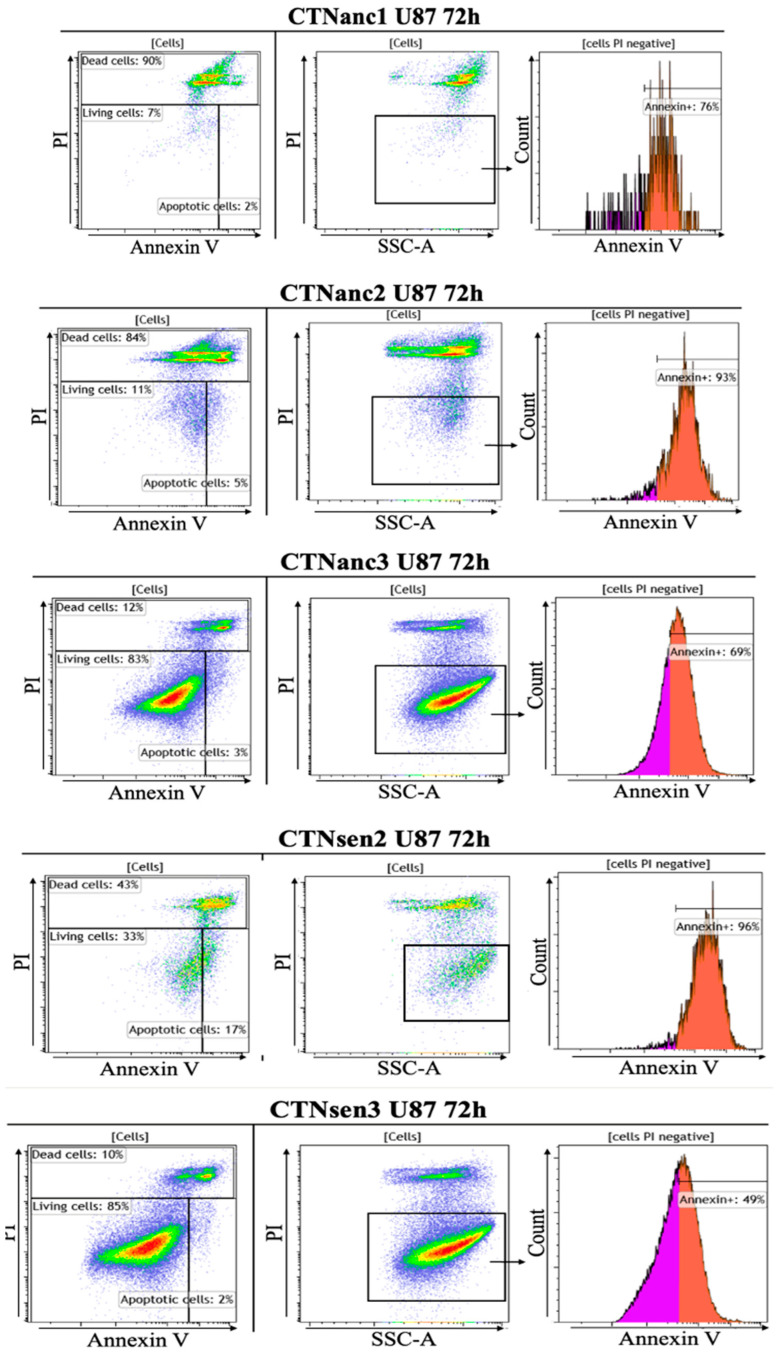
Dot plots analysis of U87 cell responses to cytotoxin peptides over 72 h by flow cytometry. Cells were labeled with Annexin-V and Propidium iodide (PI). Cells that did not undergo treatment served as the negative control, Annexin V-negative and positive cell subsets are colored in pink and orange, respectively. Annexin V-negative and positive cell subsets are colored in pink and orange, respectively on histograms.

The Annexin V-FITC/PI staining analysis (Figure 15) revealed that the peptides CTNanc1 and CTNanc2 exhibited high cytotoxicity, notably reducing the viability of U87 cells. Specifically, cells treated with CTNanc1 showed a viability of only 7%, while those treated with CTNanc2 had a viability of 11% (Table 4). In addition, compared to the control, a higher percentage of Annexin V positive cells and Mean Fluorescence Intensity (MFI) are shown for CTNanc1 and CTNanc2 in Table 5, suggesting apoptosis. In contrast, the peptides CTNanc3 and CTNsen3 demonstrated much lower cytotoxicity, with cell viability percentages of 83% and 85%, respectively, indicating that they are less toxic to U87 cells. While the MTT assay indicated activity against U87 cells at the tested concentration (1 × 10^−4^ Molar), flow cytometry did not show pronounced cytotoxic effects in terms of apoptosis or necrosis. This discrepancy could be due to several factors: (i) differential sensitivity: the MTT assay measures metabolic activity, specifically mitochondrial activity, which can decrease due to factors other than cell death, such as metabolic reprogramming, cell cycle arrest, or temporary metabolic suppression. (ii) Flow cytometry directly measures cell death by quantifying apoptotic and necrotic markers. CTNanc3 and CTNsen3 might induce a form of cell death that was not detectable by the flow cytometry assay used, such as a slower or more regulated process that does not manifest within the same timeframe as the more aggressive cytotoxic actions observed with other CTNs. These toxins might also exert effects that modulate cell behavior or viability without leading to outright cell death, impacting processes like migration, invasion, or proliferation.

Our hypothesis is that: (i) these CTNanc3 and CTNsen3 might be triggering sublethal stress responses that temporarily impair mitochondrial function or overall cellular metabolism without necessarily leading to cell death. Such stress could activate survival pathways, allowing cells to maintain viability even in the face of cytotoxic challenges. (ii) The peptides might induce autophagy, a process that can either be a survival mechanism or lead to cell death. Autophagy can reduce mitochondrial activity as part of a broader cellular recycling process, which could explain the reduced MTT signal. 

Notably, CTNsen2 also exhibited considerable cytotoxicity, reducing cell viability to 33%, though it was not as extreme as CTNanc1 and CTNanc2 when compared with the control untreated cells (Table 4 and Figure 14). However, treatment of U87 cells with CTNsen2 showed the highest Annexin V Mean Fluorescence Intensity (MFI) and 96% of the cells were Annexin V-positive (Table 5), compared to untreated cells. This suggests that CTNsen2 induces cytotoxicity leading to the apoptotic stage after 72 h.

Upon further analysis, the rise in Annexin V-positive cells within the PI-negative population (Annexin V+/PI−) suggests that early apoptosis is the primary mechanism of cell death induced by these cytotoxins, particularly for CTNanc1 and CTNanc2, which showed 76% and 93% early apoptotic cells, respectively (Table 5). These percentages reflect early apoptosis, as they indicate cells that have externalized phosphatidylserine (Annexin V positivity) but still retain membrane integrity (PI-negative).

The rise in PI-positive cells, indicative of late-stage apoptosis or necrosis, was not directly measured in the specific columns of Table 5, but the overall increase in cytotoxicity suggests that apoptosis progresses to later stages in treatments with CTNanc1 and CTNanc2.

Apoptosis and necrosis represent two distinct modalities of cellular demise [27]. Historically, necrosis was perceived as a spontaneous and unregulated form of cell death, characterized by the swelling of organelles and fragmentation of the nucleus. However, recent research has increasingly demonstrated that necrosis can also occur through a controlled process, despite being independent of caspase activation [28]. This regulated form of necrosis, often referred to as programmed necrosis or necroptosis, involves specific molecular pathways that orchestrate cellular disintegration.

In 2019, Lui et al. reported that CTX1 from *Naja atra* induces necroptosis in leukemia cells. The team initially observed a reduction in cell viability following CTX1 treatment, which was not mitigated by Z-VAD-fmk, a caspase inhibitor, suggesting a caspase-independent pathway. Crucially, pretreatment with Necrostatin-1 (Nec-1), a specific inhibitor of necroptosis, significantly restored cell viability, indicating that the mode of cell death induced by CTX1 was necroptosis. Further supporting these findings was the absence of classic apoptotic features such as chromatin condensation and nuclear fragmentation. Instead, the researchers documented early loss of plasma membrane integrity and cellular swelling, which are characteristic signs of necroptosis [16]. CTX1 (P60304 · 3SA1_NAJAT) is classified as an S-type cytotoxin, similar to CTNanc1, suggesting that the significant increase in PI-positive U87 cells may be attributed to necroptosis. To further explore this hypothesis, planning experiments using Necrostatin-1 (Nec-1), a specific inhibitor of necroptosis, would be advisable.

## 3. Conclusions

This study represents preliminary research into the cytotoxic effects of novel peptides isolated from the venoms of *Naja anchietae* and *Naja senegalensis*, marking a significant advancement in our understanding of venom cytotoxins. We successfully isolated six cytotoxins that have not previously been described in these species. Each peptide was rigorously characterized both physicochemically and pharmacologically to ascertain their potential as therapeutic agents. Two cytotoxin peptides, CTNanc1 and CTNsen3, demonstrated antiproliferative activity on U87 cell lines without exhibiting notable cytotoxicity against human umbilical vein endothelial cells (HUVECs). Our findings are particularly groundbreaking as they introduce cytotoxins as potential therapeutic agents against glioblastoma, a context in which venom-derived cytotoxins have not been extensively studied. To our knowledge, this is among the first studies to explore and document the efficacy of snake cytotoxins from these specific snakes against glioblastoma cell lines. 

## 4. Materials and Methods

### 4.1. RP-HPLC Purification

Solvents were purchased from Sigma-Aldrich (Saint-Quentin-Fallavier, France) and crude venoms of *Naja senegalensis* and *Naja anchietae* were supplied by Latoxan (Portes lès Valence, France). To purify our molecules, venoms solubilized in water at a concentration of 50 mg/mL were filtered through 0.45-micron filters. The peptides were purified from the filtrates using RP HPLC Agilent 1260 Infinity (Agilent Technologies, Santa Clara, CA, USA) on RP column Phenomenex Jupiter 300A 5 μm C18 250 mm × 21.2 mm (Phenomenex Inc., Torrance, CA, USA). The gradient used for this purification is 20% to 50% over 120 min of solvent B, which consisted of 0.1% TFA in 90% acetonitrile, and solvent A, containing 0.1% TFA in water. The fractions collected every 0.1 min were analyzed using a Shimadzu LC-2010 HPLC system (Shimadzu, Kyoto, Japan) on RP column MACHEREY-NAGEL NUCLEOSIL 100-5 C18, 5 µm, 250 × 4.6 mm (Düren, Germany).

### 4.2. Mass Spectrometry Analysis

The isolated *Naja* venom peptides were characterized by spectrometry MALDI–ToF using an α–cyanohydrocinnamic acid (HCCA) matrix (Bruker Daltonics, Wissembourg, France) (0.7–1 μL equal volumes of saturated solution at 10 μg/μL in MeCN or in 50% MeCN/0.3% TFA/H_2_O). Spectra recorded in linear positive method LP12 kDa (Bruker microflex) or HMASS 5–20 kDa were externally calibrated with suitable standards as Prot cal I of Brucker (Bruker Daltonics, Wissembourg, France) with a laser (λ = 337 nm, W = 75.2 μJ, 500 shots of υ = 10 Hz), and were analyzed by a Bruker Daltonics flex analysis software Flex Control 1.2 SP1 version 3.0 (2008) (Bruker, Palaiseau, France).

### 4.3. Reduction and Alkylation of Peptides

In total, 20 nanomoles of each peptide sample were dissolved in 160 uL of alkylation buffer containing 6 M guanidine hydrochloride (GuHCl), 0.5 M Tris/HCl, 2 mM EDTA (pH 7.5), and 4.8 μM dithiothreitol. Following 20 h of reduction in the dark, at 40 degrees, 6 μM of 4-vinylpyridine (4-VP) was added to initiate the alkylation process. The proteins that were S-alkylated were then purified using a reverse-phase C8 column (5 µm; 4.6 × 250 mm) on a RP HPLC Agilent 1260 Infinity system (Agilent Technologies, USA).

### 4.4. N-Terminal Sequencing

N-terminal sequencing of S-alkylated peptides was conducted through Edman degradation. Samples were deposited onto a glass fiber membrane GFD that had been pre-treated with 10 µL of biobrene and conditioned using the PRETREATGFD program. A calibration was performed using a solution of phenylthiohydantoin (PTH)-amino acid standard at a concentration of 10 pmol. The membrane was then inserted into the cartridge of the Shimadzu PPSQ 31B sequencer (Kyoto, Japan), and analysis was performed. The obtained sequences were subsequently cross-referenced with entries in the Uniprot database.

### 4.5. Peptide Mass Fingerprintings

Peptides were digested by AspN enzyme (Promega, Madison, WI, USA) according to manufacturer recommendations. In total, 10 µL of the sample at 7 mg/mL in H_2_O (1 µg/µL) was treated with 10 µL of 0.1 M DTT in Ammonium Bicarbonate 0.1 M for 20 min at 56 °C, then alkylated by 10 µL Iodoacetamide 10 mg/mL in Ammonium Bicarbonate 0.1 M. Then, 2 µg AspN was resuspended in 50 µL H_2_O, and 5 µL (200 ng) (1/50 *w*/*w*) was added to the reaction middle and incubated overnight at 37 °C. Peptides were then resuspended in a α-cyano-4-hydroxycinnamic acid matrix solution (saturated solution in 50% acetonitrile/0.3% TFA), and 1 µL was spotted on the Maldi metal target. Mass analyses were performed on a MALDI-TOF-TOF Ultraflex III spectrometer (Bruker Daltonics, Wissembourg, France) controlled by the Flexcontrol 3.0 package (Build 51). This instrument was used at a maximum accelerating potential of 25 kV and was operated in reflector mode and the *m*/*z* range from 600 to 3500. Six external standards (Peptide Calibration Standard II, Bruker Daltonics) were used to calibrate each spectrum to a mass accuracy within 10 ppm with a minimal resolution of 10,000 for the angiotensin II peak (Monoisotopic mass = 1,046,542 Da). Peak picking was performed with Flexanalysis 3.0 software (Bruker) with an adapted analysis method. Parameters used were as follows: SNAP peak detection algorithm, S/N threshold fixed to 6, and a quality factor threshold of 30.

### 4.6. MS/MS Analysis

The sequence of P1V214 peptide was confirmed by MS/MS analyses on the Maldi TOF-TOF Ultraflex III spectrometer (Bruker Daltonics, Wissembourg, France) using the LIFT_2017 method after calibration. LIFT mass spectra were acquired on a Bruker Ultraflex TOF/TOF mass spectrometer (Coventry, UK) operated in the positive ion mode. Metastable fragmentation was induced by a nitrogen laser (337 nm) without the further use of collision gas. Precursor ions were accelerated to 8 kV and selected in a timed ion gate. In the LIFT-cell the fragments were further accelerated to 19.07 kV. The reflector potential was 29.6 kV [29].

### 4.7. Cell Culture

U87 glioma cells and human umbilical vein endothelial cells (HUVECs) were cultured under controlled laboratory conditions. The U87, T98G and U251 cells were grown in Dulbecco’s Modified Eagle Medium (DMEM) supplied by Gibco™ (Sigma, St. Louis, MO, USA), supplemented with 10% fetal bovine serum (FBS) and 1% l-glutamine. HUVECs, procured from Lonza (Paris, France), were maintained in EBM-2 medium from the same supplier. This growth medium was fortified with hydrocortisone (1 μg/mL), bovine brain extract (12 μg/mL), epidermal growth factor (10 ng/mL), and 2% FBS (Life Technologies, Carlsbad, CA, USA). These cells were kept at 37 °C in a humidified environment containing air and 5% CO_2_. HUVECs were regularly tested and confirmed to be free from mycoplasma contamination and cultured up to the fifth passage to ensure viability and consistency in experiments. 

### 4.8. Cell Viability Assay

U87 and T98G were plated at a density of 4000 cells per well and U251 cells at a density of 2500 cells per well in microtiter plates and allowed to adhere overnight at 37 °C in a 5% CO_2_ humidified environment. The following day, the medium was replaced with fresh medium containing our venom peptides under investigation. After a 72-h incubation period, cells were treated with MTT (Promega, Lyon, France) at a concentration of 0.5 mg/mL. The MTT crystals, reduced by the cells’ mitochondrial dehydrogenases, were then solubilized using DMSO. Viable cells were then quantified by measuring the absorbance at 570 nm. HUVEC cells were plated at a density of 10,000 cells per well in microtiter plates and tested under the same conditions. Concerning the statistical analysis, modeling the differential cytotoxicity of CTNs in cancerous and non-cancerous cell lines was achieved using a log-logistic fit. Dose–response analyses were performed using the drc package [30], available from R software (R Core Team, 2023) [31]. The best results were obtained through three-parameter log-logistic fitting models after a Box-Cox transformation for each peptide/cell line combination. Model calibration was assessed using the drm() function. The half-maximal inhibitory concentration (IC50) and 95% confidence interval limits were obtained from the ED() function.

### 4.9. Flow Cytometry Analysis

U87 cells underwent flow cytometric analysis following staining with Annexin V-FITC and Propidium Iodide (PI). These cells were evaluated using a CytoFLEX S (Beckman Coulter, Brea, CA, USA) flow cytometer. For the experimental setup, cells were seeded into 6-well plates and treated with the different peptides, each at a concentration of 10^−4^ M. After a 72-h incubation period, cells were trypsinized and harvested, ensuring collection of both suspended and adherent cells. For the staining process, 5 µL of Annexin-V-fluorescein isothiocyanate (FITC) and 10 µL of propidium iodide (PI) were added to approximately 1 × 10^5^ cells. The mixture was incubated for 15 min at room temperature, shielded from light. Subsequently, the cells were immediately subjected to flow cytometric analysis. The categorization of cells was based on their staining patterns: viable (Annexin V−/PI−), early apoptotic (Annexin V+/PI−), late apoptotic or secondary necrosis (Annexin V+/PI+), and necrotic (Annexin V−/PI+).

## Figures and Tables

**Table 1:** Determined sequences by EDMAN sequencing and their homologous sequences by BLAST.

Peptide	Determined Sequence	Homologous Sequence Identified Using BLAST/UniProtKB/Swiss-Prot-% of Identity
CTNsen1	LKCHQLVPPFWKTCPEGKNLCYKMYMVSSSTVPVKRGCIDVCPKNSALVKYVCCNTDKCN	P01457.1 Cytotoxin 5 *Naja haje haje* 100%
CTNsen2	LKCHKLVPPFWKTCPEGKNLCYKMYMVATPMIPVKRGCIDVCPKNSALVKYVCCNTNKCN	P01464.1 Cytotoxin 5 *Naja haje annulifera* 98.33%
CTNsen3	LKCHQLVPPFWKTCPEGKNLCYKMYMVATPMIPVKRGCIDVCPKNSALVKYMCCNTDKCN	P01463.1 Cytotoxin 2 *Naja nivea* 98.33%
CTNanc1	LKCHKLVPPVWKTCPEGKNLCYKMFMVSTSTVPVKRGCIDVCPKDSALVKYVCCSTDKCN	P01456.1 Cytotoxin 1 *Naja nivea* 100.00%
CTNanc2	LKCHKLVPPFWKTCPEGKNLCYKMYMVATPMLPVKRGCIDVCPKDSALVKYMCCNTDK	P01462.1 Cytotoxin 2 *Naja annulifera* 100.00%
CTNanc3	LKCHKLIPPFWKTCPEGKNLCYKMYMVATPMIPVKRGCIDVCPKDSALVKYMCCNTDKCN	P01463.1 Cytotoxin 2 *Naja nivea* 98.33%

**Table 2:** Some examples of studies of Type S and Type P CTXs derived from *Naja* species against cancer.

Cytotoxin	Type	Active Against/Dose	Venom Source	Reference
CTX-2N	Type S	0.8 μM for A549 non-small cell lung adenocarcinoma	*Naja nigricollis*	Conlon et al., 2020 [14]
NK-CTX/Cytotoxin 3	Type S	Lung cancer (A549) A549 = 1.22 μg/mL Prostate cancer PC-3 = 4.46 μg/m Breast cancer MCF-7 = 12.23 μg/mL	*Naja kaouthia*	Chong et al., 2020 [2]
CTX-II	Type P	Breast cancer (MCF-7)	*Naja oxiana*	Ebrahim et al., 2014 [15]
Cytotoxin 2a/NS-CTX	Type P	Lung cancer (A549) 0.88 μg/mL Prostate cancer PC-3 = 3.13 μg/m Breast cancer MCF-7 = 9.10 μg/mL	*Naja sumatrana*	Chong et al., 2020 [2]
Cytotoxin 1	Type S	Acute myeloid leukemia (KG-1a) 3.31 μg/mL	*Naja atra*	Liu et al. (2019) [16]
Cytotoxin 3/NN-32	Type S	Breast cancer MCF-7 = 2.5 μg/mL	*Naja naja*	Attarde and Pandit (2017) [17]
CT1No	Type S	Leukemia WEHI-3 = 340 nM Myelogenous leukemia K562 = 650 nM Acute promyelocytic leukemia HL-60 = 1400 nM	*Naja oxiana*	Feofanov et al. (2004) [18]
CT2Nh	Type P	Leukemia WEHI-3 = 1190 nM Acute promyelocytic leukemia HL-60 = 3700 nM	*Naja haje*	Feofanov et al. (2004) [18]
Cytotoxin 3	Type P	Human neuroblastoma SK-N-SH = 0.8 µM Breast adenocarcinoma MDA-MB-231 = 0.09–0.15 mM	*Naja atra*	Chen et al. (2008) [19], Lin et al. (2010) [20]
Cytotoxin 4	Type S	Human neuroblastoma SK-N-SH = 1.2 µM	*Naja atra*	Chen et al. (2008) [19]

**Table 3:** Differential cytotoxicity of CTN’s in cancerous and non-cancerous cell lines. The half maximal inhibitory concentration (IC50) was estimated using a three-parameter log-logistic fit along with 95% confidence intervals limits (lower, higher). * Non-cytotoxic activity at 1 × 10^−4^ Molar.

Tissue Origin	Cell Line	IC50 (μg/mL)					
		CTNanc1	CTNanc2	CTNanc3	CTNsen1	CTNsen2	CTNsen3
Brain Glioblastoma	U87	36.41	38.34	5.33	54.69	Inactive	45.38
95% confidence interval		32.87–39.96	34.63–42.04	4.65–6.02	46.11–63.28		36.80–53.97
Brain Glioblastoma	U251	Inactive	475.8	416.37	Inactive	Inactive	Inactive
95% confidence interval			395.04–556.56	338.15–494.59			
Brain Glioblastoma	T98G	Inactive	389.59	420.01	Inactive	462.18	Inactive
95% confidence interval			296–483.18	384.96–455.06		386.35–538.01	
Endothelial	HUVEC	Inactive *	467.21	3.58	225.65	Inactive *	Inactive *
95% confidence interval			423.44–510.97	3.42–3.74	167.03–284.28		

**Table 4:** Data from the initial gating strategy show cellular responses after 72 h of treatment with cytotoxin peptides at a concentration of 1 × 10^−4^ Molar. The percentages of living and dead cells reflect the effects of various cytotoxin peptides on U87 cell samples.

Sample Tested	% Living Cells Gated	% Apoptotic Cells Gated	% Dead Cells Gated
**Control U87 cells**	81	2	14
**CTNanc1-U87**	7	2	90
**CTNanc2-U87**	11	5	84
**CTNanc3-U87**	83	3	12
**CTNsen2-U87**	33	17	43
**CTNsen3-U87**	85	2	10

**Table 5:** Data from the second part of the gating strategy focus on the analysis of apoptotic markers in PI-negative U87 cells treated with cytotoxin peptides for 72 h at a concentration of 1 × 10^−4^ Molar. The Mean Fluorescence Intensity (MFI) of Annexin V-FITC and the percentage of Annexin V-positive cells, derived from histograms, indicate the extent of apoptosis in each treatment group.

Sample Tested	[Cells PI Negative Cells] Number	[Cells PI Negative Cells] Annexin FITC MFI	[AnnexinV^+^] % Gated from Histograms
**Control U87 cells**	71,033	29,764	50
**CTNanc1-U87**	312	59,862	76
**CTNanc2-U87**	2737	164,227	93
**CTNanc3-U87**	68,626	46,465	69
**CTNsen2-U87**	3689	200,997	96
**CTNsen3-U87**	65,486	29,041	49

**Figure A2:**
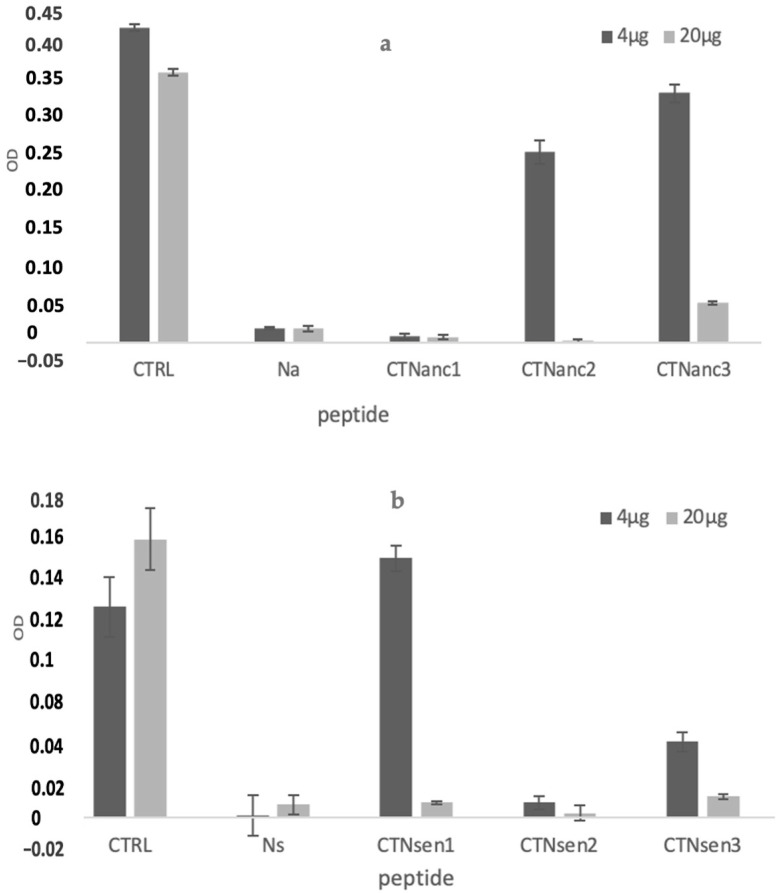
Antiproliferative activity of CTNsen1, CTNsen2, CTNsen3, CTNanc1, CTNanc2, and CTNanc3 from *Naja senegalensis* (**b**) and *Naja anchietae* (**a**), respectively. These results were obtained using an MTT assay on MCF7 cells after 72 h of treatment at 4 µg and 20 µg of peptide. Na and Ns here were used as a positive control at two concentrations (3 µg and 15 µg). The OD was measured at 570 nm.

## Data Availability

The results of this study are fully documented and presented within the confines of this article.

## Appendix A

Through meticulous HPLC fractionation, we were able to isolate six distinct peptides, named CTNsen1, CTNsen2, CTNsen3, CTNanc1, CTNanc2, and CTNanc3, from *Naja senegalensis* and *Naja anchietae*, respectively. Their activity was confirmed on MCF7 cells as shown in Figure A2.
